# A retrospective cohort study on retrograde intramedullary nailing in management of femur fractures in tertiary care hospital at Bujumbura, Burundi

**DOI:** 10.1097/MD.0000000000042913

**Published:** 2025-06-27

**Authors:** Fabrice Kibukila, Jean Claude Niyondiko, Gilbert Ndayizeye, Moise Cuma Zihindula, Gilbert Ndayishimiye, Patrick Hakizimana, Soham Bandyopadhyay, Calvin R. Wei, Samuel A. Mbabazi, Rodrigue Mupenda Mwenibamba, Aymar Akilimali

**Affiliations:** a Faculty of Medicine, University of Burundi, Bujumbura, Burundi; b Faculty of Medicine, Official University of Bukavu, Bukavu, DR Congo; c Faculty of Medicine, Université Paris Cité, Paris, France; d Clinical Neurosciences, Clinical and Experimental Sciences, Faculty of Medicine, University of Southampton, Southampton, Hampshire, United Kingdom; e Oxford University Global Surgery Group, Nuffield Department of Surgical Sciences, University of Oxford, Oxford, United Kingdom; f Department of Research and Development, Shing Huei Group, Taipei, Taiwan; g Faculty of Medicine, University of Goma, Goma, DR Congo; h Department of Surgery, HEAL Africa Tertiary Hospital, Goma, DR Congo; i Department of Research, Medical Research Circle (MedReC), Bukavu, DR Congo.

**Keywords:** Burundi, femoral fractures, retrograde intramedullary nailing

## Abstract

Intramedullary nailing happens to be the standard treatment for fractures. The purpose of this project is to weigh the place of retrograde intramedullary nailing in the management of femoral fractures in a surgical setting of a low-income country and to analyze the radiological and functional outcome obtained after this type of osteosynthesis. We carried out a cohort and analytical study over a period of 10 years, period between 2013 and 2023, on a total of 140 persons who experienced retrograde intramedullary nailing indicated for femur fracture in the orthopedic and traumatology department of the surgical department of a tertiary care hospital in Bujumbura, Burundi. Retrograde femoral nailing involved 27.72% (140 of 505) of patients who were treated with Centro medullary femoral nailing in general. The mean age of the patients was 42.30 years, where road accidents accounted for 77.85% of the causes of trauma. Fractures classified as AO 32C3 were in the majority (24.29%). The age of the patients was not statistically associated with knee pain (*P* = .61), nor with knee flexion (*P* = .33). However, we found a correlation between the patients’ origin and knee pain (*P* = .01), independence of movement (*P* < .01) and knee flexion (*P* = .03). Retrograde femoral nailing remains a therapeutic method that can be used in both elderly and young subjects with satisfactory medium-term functional results.

**Key points**

- Femoral fractures are a significant global health issue – representing approximately 9% of injuries presenting as surgical emergencies – that often result from road traffic collisions.
- In low-income settings such as Burundi, managing these injuries presents unique challenges due to resource constraints.
- Retrograde intramedullary nailing (RIN) has emerged as a favored technique. However, the application and outcomes of this procedure in resource-limited settings require further exploration.

## 1. Introduction

Currently, femur fractures represent approximately 9% of injuries admitted to surgical emergencies and are largely related to road traffic accidents.^[1]^ In the current scenario of the surgical field, treatment options for these fractures have become incrementally diverse and have established its efficacy in low-income settings.^[2]^ Intramedullary nailing is the reference surgical treatment for femur fractures in adults.^[3]^ During the past few days, Küntcher’s simple intramedullary nailing was increasingly abandoned because it did not provide rotational control or overlapping fragments in complex fractures. The addition of a nail-to-bone interlock, described by Green in 1970,^[4]^ provides rotational stability and avoids impaction of the bone fracture site.^[5]^ Retrograde intramedullary nailing remains the technique of choice for femoral diaphyseal fractures, particularly in its middle and distal thirds.^[6]^ Not only does it stabilize the fractures of these segments, but the approach used also makes it possible to fix the associated bone lesions (fractures of the patella, of the ipsilateral tibia).^[7]^ As with other minimally invasive techniques, preservation of local vasculature and soft tissue improves outcomes and reduces complication rates associated with open reduction and internal fixation, making nailing a therapeutic choice for femoral fractures, especially in the elderly.^[4,8]^ Femoral fractures by retrograde femoral nailing pose a problem of indication. In the United States of America, retrograde femoral nailing is reserved for patients of all ages,^[9,10]^ and in European countries, elderly patients and patients with knee fractures seem to be increasingly concerned.^[8,11]^

A disadvantage of retrograde femoral nailing is that correct limb length, axial alignment, and rotation must be assessed by means other than open reduction. Some authors have reported a rotational misalignment after intramedullary nailing of up to 28%, resulting in aesthetic and functional disability and to avoid this drawback, the surgeon must be attentive to the malrotation, because a subsequent intervention is the only way. to correct the deformation once the nail is locked.^[12]^ Despite a good rate of bone healing,^[13]^ retrograde nailing is associated with complications, including knee pain and stiffness, which should be monitored in patients.^[14]^ The objective of this study is to evaluate the place of retrograde intramedullary nailing in the management of femoral fractures in a surgical context of a low-income country and to analyze the radiological and functional results obtained after this type of osteosynthesis.

## 2. Methods

### 2.1. Study setting and design

This is a cross-sectional and retrospective study of a total of 140 patients who underwent retrograde intramedullary nailing, indicated for a femur fracture in the surgery department of the tertiary care hospital of Bujumbura in Burundi. Over a period of 10 years, i.e., a period from January 2013 to January 2023.

### 2.2. Data collection and study variables

After approval by the university ethics committee and authorization from the local committee to collect the data within their hospital. The epidemiological and clinical data, the data on the operating and radiographic methods were recorded on the Surgical Implant Generation Network Surgery software (SIGN Surgery software). All data was collected in a Microsoft Excel 2016 sheet and then analyzed in IBM SPSS 2020 software for statistical analysis.

Several variables were studied, including epidemiological variables (age, sex, origin, cause of trauma and operating time in patients), clinical variables (the side of the fractured bone, the fracture site, the type of line, the number of bone fragments, the Association of Osteosynthesis (AO) classification, the types of fracture according to the cutaneous opening, the presence of another anterior osteosynthesis, the types of anterior osteosynthesis, the notion of reaming, the types of reduction, nail types, nail length, nail diameter, numbers of proximal and distal screws) and variables that are related to patient outcome (pain, gait and knee stiffness).

### 2.3. Statistical analysis and postoperative follow-up

All the patients included in this study were followed up initially 4 weeks following the surgical intervention, then every 3 months for the control in order to observe the evolution until complete healing (presence of a consolidation callus on the X-ray).

It should be noted that 42 of the patients (30% of the total number) benefited from specific postoperative follow-up (for indefinite periods). The Sikorski and Barrington score^[15,16]^ (Table 1) and knee flexion looking for joint stiffness were assessed for functional outcome assessment. Data were entered into Microsoft Excel 2016 and then transferred to Stata SE 14.0 (Stata Corp LP, College Station, Texas, USA) for cleaning and analysis. To describe the data, some quantitative variables were categorized and analyzed as qualitative variables summarized as frequencies and percentages. To compare proportions, we used Pearson chi-square test or Fisher exact test for proportions less than or equal to 5. We constructed univariate and multivariate logistic regression models to assess the factors associated with patients’ functional follow-up after intervention (pain, knee flexion and walking). The odds ratio and their 95% confidence intervals were calculated to measure the strength of the association between the variables. To integrate the final model, we considered values of *P* < .2 and 0.05. All p-values were 2-tailed and a *P*-value < 0.05 was used as the significance level.

**Table 1:** Sikorski and Barrington score.^[15,16]^

Grade	Evaluation
*Pain*	
1	No pain
2	Occasional pain, no analgesics
3	Occasional or constant pain with occasional analgesics
4	Constant and severe pain with regular use of analgesics
*Walk*	
1	Independent patient, walks unaided, can do errands and able to use public transportation
2	Independent patient, walks unassisted, can-do errands and is able to use public transportation but requires aids
3	Limited movement in the house without assistance
4	Movement is limited in the house but with aids
5	Patient can sit up
6	Patient remains in bed

The technique proposed by SIGN Surgery software was used for approaching the femur.^[17]^ The patient was installed in the supine position on a standard table. Nonconstant knee flexion at 60° was performed using a sub-popliteal support. A closed reduction can be attempted with or without scope control; in case of failure, an open reduction was performed through a lateral incision. In the absence of intra-articular fracture, a medial parapatellar or trans-patellar incision is made while it remains medial parapatellar in the presence of it. The nail was inserted medially into the intercondylar notch at the osteochondral junction. Transverse screws were placed to stabilize the fracture after insertion.

### 2.4. Ethics approval and consent to participate

Ethical approval for this study (Ethical Committee UnBur/FacMed/00197/DR/2021) was provided by the Ethical committee of the University of Burundi, Burundi on April 15th, 2022. The study design incorporated the separation of patient identification data by confidentiality codes to maintain anonymity and protect the privacy of the participants. This study was performed in accordance with the ethical standards as laid in the 1964 Declaration of Helsinki and its later amendments or comparable ethical standards.

## 3. Results

Retrograde femoral nailing was performed in 140 patients, or 27.72% (140 out of 505) of patients treated by intramedullary femoral nailing in general. The average age of the patients was 42.30 years and 42.14% of our patients lived in urban areas, the male sex was the most represented with 62.85% and a sex ratio was around 1.7/ 1, the average duration of operation was 133.01 days, road traffic accidents represent 77.85% of the causes of trauma in our patients.

The clinical data have been represented in Table 2. The left femur was the most affected with 52.86% and the distal segment being the most affected with 55.71%. In 58.57% of cases, the fractures were simple and with 2 fragments, the transverse line being the most frequent with 45.00%, fractures classified AO 32C3 were the majority with 24.29%. The fractures were closed in 89.29% of the cases and the reduction was open in 87.14%, the standard nails were used most often in 71.42% of the cases and the nails of 360 cm in length and 9 cm diameter were most often used and represent respectively 41.42% and 49.28%, double locking of screws (proximal and distal) was achieved in 79.28% of patients.

**Table 2:** Clinical characteristics of participants.

Variables		N (140)	Percentage (%)
Fractured femur side			
Right		66	47.14
Left		74	52.86
Fracture site			
Proximal third		4	2.86
Average third party		58	41.43
Distal third		78	55.71
Type of line			
Spiral		31	22.14
Oblique		46	32.86
Transversal		63	45.00
Number of fragments			
Two fragments		82	58.57
Three fragments		18	12.86
More than 3 fragments		31	22.14
Bifocal		9	6.43
Fracture according to the skin opening			
Open fracture		15	10.71
Closed fracture		125	89.29
AO Classification			
1/3 medium (N = 127)	A1	23	16.43
	A2	24	17.14
	A3	26	18.57
	B2	6	4.29
	B3	6	4.29
	C2	8	5.71
	C3	34	24.29
1/3 low (N = 13)	A2	4	2.86
	A3	8	5.71
	C2	1	0.71

AO = association of osteosynthesis.

Starting from the results related to the postoperative follow-up of the patients, the results have been represented in Table 3. Of the 42 patients followed postoperatively, 16 (38.10%) of them reported no knee pain and 26 (61.90%) other patients felt independent and walked without help or support. they can do their shopping and they can also use public transport. Knee flexion was assessed between 90 and 150° in 33 (78.57%) of the 42 patients followed postoperatively.

**Table 3:** Postoperative follow-up of patients.

Variables	N (42)	Percentage (%)
Pain		
Grade 1	16	38.10
Grade 2	10	23.81
Grade 3	14	33.33
Grade 4	2	4.76
Walk		
Grade 1	26	61.90
Grade 2	11	26.19
Grade 3	4	9.52
Grade 4	1	2.38
Knee flexion		
Less than 90°	9	21.43
Between 90° and 150°	33	78.57

For the data on the distribution of patients related to pain in follow-up and clinic epidemiological characteristics (Table 4). Of the patients who had postoperative follow-up, 38.89% of them were between the ages of 20 and 39 and did not report pain, the same was true for 50.00% of patients having developed fractures classified as AO 32A2; however, the differences were not statistically significant (*P* = .61 and *P* = .74 respectively). In contrast, we found a statistically high proportion (64.71%) of patients from urban areas who did not report pain at follow-up (*P* = .01).

**Table 4:** Distribution of patients related to pain in follow-up and clinic epidemiological characteristics.

Variables (N = 42)	Pain				*P*-value
	Grade 1, n (%)	Grade 2, n (%)	Grade 3, n (%)	Grade 4, n (%)	
Age range					
<20 yr	2 (40.00)	1 (20.00)	2 (20.00)	0 (0.00)	.61
20 to 39 yr old	7 (38.89)	6 (38.33)	4 (22.22)	1 (5.56)	
40 to 59 yr old	6 (50.00)	1 (8.33)	5 (41.67)	0 (0.00)	
>60 yr old	1 (14.29)	2 (28.57)	3 (42.86)	1 (14.29)	
Provenance					
Urban	11 (64.71)	3 (17.65)	2 (11.76)	1 (5.88)	**.01**
Semi-urban	1 (20.00)	1 (20.00)	2 (40.00)	1 (20.00)	
Rural	4 (20.00)	6 (30.00)	10 (50.00)	0 (0.00)	
AO classification (AO 32)					
A1	2 (25.00)	3 (37.50)	2 (25.00)	1 (12.50)	.74
A2	5 (50.00)	2 (20.00)	3 (30.00)	0 (0.00)	
A3	3 (50.00)	2 (20.00)	3 (30.00)	0 (0.00)	
B2	1 (33.33)	0 (0.00)	1 (33.33)	1 (33.33)	
B3	2 (66.67)	0 (0.00)	1 (33.33)	0 (0.00)	
C2	0 (0.00)	2 (66.67)	1 (33.33)	0 (0.00)	
C3	3 (20.00)	1 (20.00)	3 (60.00)	0 (0.00)	

The bold value indicates significance at *P* < .05.

AO = association of osteosynthesis.

Table 5 represents the distribution of the patients according to the gait at the postoperative follow-up and their clinical and epidemiological characteristics. Mobility independence was significantly high in patients from urban areas (*P* < .01). On the other hand, we did not find a statistically significant difference between walking with age group (*P* = .87) and AO classification (*P* = .11).

**Table 5:** Distribution of patients in relation to walking in follow-up and clinic epidemiological characteristics.

Variables (N = 42)	Walk				*P*-value
	Grade 1, n (%)	Grade 2, n (%)	Grade 3, n (%)	Grade 4, n (%)	
Age range					
<20 yr old	4 (80.00)	1 (20.00)	0 (0.00)	0 (0.00)	.87
20–39 yr old	11 (61.11)	5 (27.78)	2 (11.11)	0 (0.00)	
40–59 yr old	8 (66.67)	3 (25.00)	1 (8.33)	0 (0.00)	
>60 yr old	3 (42.90)	2 (28.57)	1 (14.29)	1 (14.29)	
Provenance					
Urban	11 (55.00)	8 (40.00)	1 (5.00)	0 (0.00)	**<.01**
Semi-urban	1 (20.00)	1 (20.00)	3 (7.1)	0 (0.00)	
Rural	14 (82.35)	2 (11.76)	0 (0.00)	1 (5.88)	
AO classification (AO 32)					
A1	5 (62.50)	2 (25.00)	0 (0.00)	1 (12.50)	.11
A2	8 (80.00)	2 (20.00)	0 (0.00)	0 (0.00)	
A3	8 (80.00)	2 (20.00)	0 (0,00)	0 (0.00)	
B2	1 (33.33)	1 (33.33)	1 (33.33)	0 (0.00)	
B3	2 (66.67)	0 (0.00)	1 (33.33)	0 (0.00)	
C2	1 (33.33)	2 (66.67)	0 (0.00)	0 (0.00)	
C3	1 (20.00)	2 (40.00)	2 (40.00)	0 (0.00)	

The bold value indicates significance at *P* < .05.

AO = association of osteosynthesis.

In Table 6, we have the distribution of patients according to knee flexion at postoperative follow-up. We noted a statistically significant difference between the origin and the stiffness of the knee in flexion (*P* = .03). On the other hand, we did not note any statistically significant difference between knee stiffness according to age group (*P* = .33) and AO classification (*P* = .17). In Tables 7, 8, and 9, univariate and multivariate analyses were performed in relation to the patients’ clinical follow-up and showed a statistically significant association between the patients’ origin and pain (*P* = .04), knee flexion (*P* < .01) and walking (*P* = .02).

**Table 6:** Distribution of patients in relation to knee flexion in follow-up and clinic epidemiological characteristics.

Variable (N = 42)	Knee flexion		*P*-value
	Less than 90, n (%)	Between 90° and 150°, n (%)	
Age range			
<20 yr old	0 (0.00)	5 (100.00)	.33
20–39 yr old	3 (16.67)	15 (89.33)	
40–59 yr old	3 (25.00)	9 (75.00)	
>60 yr old	3 (42.86)	4 (57.14)	
Provenance			
Urban	1 (5.88)	16 (94.12)	**.03**
Semi-urban	3 (60.00)	2 (40.00)	
Rural	5 (25.00)	15 (75.00)	
AO classification (AO 32)			
A1	3 (37.50)	5 (62.50)	.17
A2	1 (10.00)	9 (90.00)	
A3	0 (0.00)	8 (100,00)	
B2	1 (33.33)	2 (66.67)	
B3	1 (33.33)	2 (66.67)	
C2	1 (33.33)	2 (66.67)	
C3	2 (40.00)	5 (60.00)	

The bold value indicates significance at *P* < .05.

AO = association of osteosynthesis.

**Table 7:** Univariate and multivariate analysis of factors associated with knee stiffness (flexion).

Variables (N = 42)	COR (CI 95%)	*P*-value	AOR (CI 95%)	*P*-value
Age range				
<20 yr old	1 (reference)		1 (reference)	
20–39 yr old	0.26 (0.03–1.86)	.18	0.07 (0.04–1.03)	.05
40–59 yr old	0.44 (0.06–3.24)	.42	0.24 (0.02–2.40)	.22
>60 yr old	–	–	–	–
Provenance				
Urban	1 (reference)		1 (reference)	
Semi-urban	24.00 (1.61–356.63)	.02	125.12 (3.41–458.92)	<.01
Rural	5.33 (0.55–51.09)	.14	5.55 (0.47–65.43)	.173
AO Classification				
A1	1 (reference)		1 (reference)	
A2	0.18 (0.01–13.63)	.18	0.16 (0.01–2.28)	.18
A3	–	–	–	–
B2	0.83 (0.05–13.63)	.89	0.32 (0.08–11.45)	.53
B3	0.83 (0.05–13.63)	.89	0.57 (0.09–35.85)	.79
C2	0.83 (0.05–13.63)	.89	0.32 (0.08–11.45)	.53
C3	1.11 (0.11–10.98)	.92	0.56 (0.04–7.56)	.66

AO = association of osteosynthesis, AOR *=* Adjusted odd ratios, CI *=* Confidence interval, COR *=* Cruds odd ratios.

**Table 8:** Univariate and multivariate analysis of factors associated with pain.

Variables (N = 42)	COR (CI 95%)	*P*-value	AOR (CI 95%)	*P*-value
Age range				
<20 yr old	1 (reference)		1 (reference)	
20–39 years old	0.57 (0.07–4.55)	.60	0.36 (0.03–3.89)	.40
40–59 years old	1.07 (0.12–8.97)	.94	0.72 (0.62–8.47)	.80
>60 yr old	2.00 (0.19–20.61)	.56	1.91 (0.13–26.79)	.63
Provenance				
Urban	1 (reference)		1 (reference)	
Semi-urban	7.00 (0.79–61.97)	.08	10.85 (1.04–112.56)	.04
Rural	4.66 (1.01–21.42)	.04	5.37 (1.03–27.96)	.04
AO Classification				
A1	1 (reference)		1 (reference)	
A2	0.71 (0.09–5.11)	.18	1.66 (0.10–25.50)	.71
A3	0.71 (0.09–5.11)	.18	1.08 (0.05–19.72)	.96
B2	3.33 (0.20–54.53)	.39	4.98 (0.21–116.54)	.31
B3	0.83 (0.05–13.63)	.89	1.45 (0.04–45.00)	.83
C2	0.83 (0.05–13.63)	.89	1.14 (0.02–58.43)	.94
C3	2.50 (0.25–24.71)	.43	5.00 (0.29–84.06)	.26

AO = association of osteosynthesis, AOR *=* Adjusted odd ratios, CI *=* Confidence interval, COR *=* Cruds odd ratios.

**Table 9:** Univariate and multivariate analysis of factors associated with walking.

Variables (N = 42)	COR (CI 95%)	*P*-value	AOR (CI 95%)	*P*-value
Age range				
<20 yr	1 (reference)			
20–39 yr old	0.74 (0.05–9.87)	.82		
40–59 yr old	0.54 (0.02–10.36)	.94		
>60 yr old	–	–		
Provenance				
Urban	1 (reference)		1 (reference)	
Semi-urban	24.00 (1.61–356.63)	.02	24.00 (1.61–356.63)	.02
Rural	–	–	–	–
AO Classification				
A1	1 (reference)			
A2	–	–		
A3	–	–		
B2	3.50 (0.11–84.69)	.44		
B3	3.50 (0.11–84.69)	.44		
C2	–	–		
C3	1.75 (0.01–1.16)	.71		

AO = association of osteosynthesis.

## 4. Discussion

Retrograde intramedullary nailing remains one of the preferred alternatives in the treatment of femoral fractures, particularly in the middle and distal thirds.

In our study, the average age was 42.30 years, the male gender was the most represented (62.9%), the average operative time was 133.01 days. The average age of our patients is comparable to some American and Indian studies where it was also relatively young (38.03 years).^[9,10,18]^ On the other hand, it is much lower than that found in the European series.^[8,11]^ This difference in average age is explained by the difference in life expectancy between our population and the European population in general, and also reinforces the problem of indications for retrograde femoral intramedullary nailing, reserved for elderly osteoporotic patients in Europe. The predominance of men in our study is explained by the fact that many drivers (motorcycle or vehicle drivers) are men, which exposes them to road traffic accidents, which is the largest cause of femur fractures. in our study. As for the average surgical time of 133.01 days, several factors may be incriminated, including the use of traditional medicine, referral often late and in the face of complications and the precariousness of the economic standard of living of the population.

All these factors have a common denominator, the delay in surgical management due to its late use. Compared to clinical data, 58.57% of fractures were simple with 2 fragments, the most frequent transverse fracture (45.00%), and the majority were fractures classified as AO 32C3 (24.29%). Our results are almost similar to those of Neubauer et al, who also report the predominance of simple fractures in their Austrian and Swiss series, but the majority are classified as AO 33 (58.3%).^[8]^ Iyengar et al in their study in the United Kingdom found a predominance of AO 32A2 fractures (7 out of 16 patients),^[6]^ whereas those classified as 32A3 were in the majority in the study by Kim et al in South Korea.^[3]^ The predominance of AO 32C3 fractures in our study would be related to the fact that most of them were high-energy injuries, with road accidents being the most frequent cause. In the postoperative follow-up, 3 parameters were evaluated, including knee stiffness, pain in the operated limb and whether or not it was independent of walking. These parameters were chosen because they are among the complications of retrograde femoral nailing.^[6,18,19]^

Of the 42 patients who underwent postoperative follow-up, 38.10% reported no pain in the operated limb; 61.90% felt independent in their movements and 78.57% were able to bend the knee between 90 and 150°. Several authors before us have also analyzed the evolution of patients who have undergone retrograde femoral nailing, and the results have been mostly satisfactory. In a study carried out in India by Acharya et al, report: 26 of 27 patients followed postoperatively were able to flex their knee beyond 100°, 77% of patients had an excellent functional score (evaluated by the Knee Society Score).^[18]^ In Austria and Switzerland, Neubauer et al found in their study that 77.3% of patients did not report knee pain during the operation.^[8]^ Many other studies have assessed the parameters mentioned above during the course of the disease, and the results were mostly acceptable.^[20,21]^ 4.76% of patients experienced constant pain requiring the regular use of analgesics; nevertheless, the presence or absence of pain in the postoperative follow-up was not statistically influenced by the age group (*P* = .61). Shah et al found data similar to our results in their work in the United States; knee pain was reported in 23% of cases, but was not statistically associated with age (*P* = .94).^[4]^ We noted a statistically significant difference between the origin of patients suffering from pain (*P* = .01), independence of movement (*P* < .01) and knee flexion (*P* = .03), this is explained by the precocity of the surgical treatment that patients in urban areas benefit from, unlike patients in semi-urban and rural areas, the faster and earlier the treatment, the better the outcome.

However, our study has some drawbacks. The first is the difficulty of accessing all patients for postoperative follow-up, only 42 (30%) were accessible, making the sample unrepresentative. The second drawback is that this study did not examine the other complications of retrograde femoral nailing described in the literature, including angular and rotational defects.^[4,12]^

We hope that future studies will take into account all these parameters to allow the future scientific generation to have almost complete data on retrograde femoral nailing in our environment.

## 5. Conclusion

Retrograde femoral nailing remains an important alternative in the treatment of femoral fractures. It is a therapeutic method that can be used in both elderly and young patients, which is a major asset in countries with limited resources such as ours, given its technical and financial accessibility which is no longer questioned. cause. Knowing how to anticipate the various complications, in particular knee stiffness, pain, angular and rotational deformities, remains a major challenge for any orthopedic surgeon in the postoperative follow-up which must be regular.

## Acknowledgments

The authors would like to thank the direction of research and the STAFF of Medical Research Circle (MedReC) from Democratic Republic of the Congo for the realization of this present paper.

## Author contributions

**Conceptualization:** Fabrice Kibukila, Jean Claude Niyondiko, Gilbert Ndayizeye, Moise Cuma Zihindula, Soham Bandyopadhyay, Calvin R. Wei, Samuel A. Mbabazi, Aymar Akilimali.

**Data curation:** Fabrice Kibukila, Moise Cuma Zihindula, Gilbert Ndayishimiye, Patrick Hakizimana, Soham Bandyopadhyay, Calvin R. Wei, Samuel A. Mbabazi, Rodrigue Mupenda Mwenibamba, Aymar Akilimali.

**Formal analysis:** Fabrice Kibukila, Moise Cuma Zihindula, Soham Bandyopadhyay, Calvin R. Wei, Samuel A. Mbabazi, Aymar Akilimali.
